# *Agrobacterium*-mediated genetic transformation of the most widely cultivated superior clone *Eucalyptus urophylla* × *E. grandis* DH32-29 in Southern China

**DOI:** 10.3389/fpls.2022.1011245

**Published:** 2023-01-17

**Authors:** Xiaoping Wang, Shanshan Chen, Haonan Zhang, Ping Luo, Fangping Zhou, Bingshan Zeng, Jianmin Xu, Chunjie Fan

**Affiliations:** ^1^ State Key Laboratory of Tree Genetics and Breeding, Chinese Academy of Forestry, Beijing, China; ^2^ Key Laboratory of State Forestry and Grassland Administration on Tropical Forestry, Research Institute of Tropical Forestry, Chinese Academy of Forestry, Guangzhou, China; ^3^ State Key Laboratory of Tree Genetics and Breeding, Northeast Forestry University, Harbin, China; ^4^ College of Life Science, Northeast Forestry University, Harbin, China

**Keywords:** *Eucalyptus*, regeneration, adventitious bud, *Agrobacterium tumefaciens*, genetic transformation

## Abstract

*Eucalyptus*, as an economically important species for wood and paper industries, remains a challenge to genetic improvement by transgenic technology owing to the deficiency of a highly efficient and stable genetic transformation system, especially in cultivated superior clones. *Eucalyptus urophylla* × *E. grandis* clone DH32-29 is most widely planted in southern China, but it is relatively recalcitrant to adventitious bud regeneration, which blocks the establishment of a genetic transformation system. Here, an efficient adventitious bud regeneration and transformation system of *Eucalyptus* was established using *E. urophylla* × *E. grandis* DH32-29 as material. The *in vitro* leaves from microshoots that were subcultured for 20–25 days were immersed into liquid Woody Plant Medium supplemented with 0.02 mg·L^−1^ α-naphthaleneacetic acid (NAA) and 0.24 mg·L^−1^ forchlorfenuron [callus-inducing medium (CIM)]. After 15 days, explants were transferred to a medium containing 0.10 mg·L^−1^ NAA and 0.50 mg·L^−1^ 6-benzyladenine (shoot-inducing medium, SIM) for adventitious bud induction. The highest regeneration efficiency of adventitious buds was 76.5%. Moreover, an *Agrobacterium tumefaciens*-mediated genetic transformation system was optimized. The leaves were precultured for 7 days and infected for 30 min with *A. tumefaciens* strain EHA105 grown to a bacterial density of 0.3 (OD_600_). After 72 h of cocultivation in the dark, leaves were transferred to CIM supplemented with 100 mg·L^−1^ cefotaxime (Cef), 100 mg·L^−1^ timentin, and 15 mg·L^−1^ kanamycin (Kan) for 15 days to induce calluses. Then, the explants were transferred to SIM supplemented with the same concentration of antibiotics, and the fresh medium was replaced every 15 days until resistant adventitious buds appeared. After inducing roots in root-inducing medium supplemented with 200 mg·L^−1^ Cef and 75 mg·L^−1^ Kan, completely transgenic plants were obtained. Using the aforementioned method, the transformation frequency can reach 1.9%. This provides a powerful approach for genetic improvement of *E. urophylla* × *E. grandis* DH32-29 and gene function analysis in *Eucalyptus*.

## 1 Introduction

*Eucalyptus* is an important multipurpose woody plant that, along with other members of the Myrtaceae including *Corymbia* and *Angophora*, originated in or near Australia (Hiwale, 2015). There are more than 700 species of *Eucalyptus*, and some are widely distributed in over 30 countries around the world and account for 16% of forest plantation areas (Teulieres and Marque, 2007). The *Eucalyptus* species are widely used in paper pulp, furniture, plywood, fuelwood, and essential oil for desirable characteristics. Due to its high economic reward, *Eucalyptus* has attracted attention from forest workers, and the plantation area covers approximately 20 million hectares after many years of introduction and afforestation (Girijashankar, 2011). In China, for example, the area planted in *Eucalyptus* was 5.47 million hectares according to the ninth National Forest Inventory (2014–2018) (National Forestry and Grassland Administration, 2019).

With the increase in single clone plantations of *Eucalyptus*, an incidence of pests occurred (Ji et al., 2011), such as *Leptocybe invasa* (Luo et al., 2014), *Ophelimus bipolaris* (Chen et al., 2021), and *Buzura suppressaria*, and led to growth reduction and tree death and caused serious yield reduction. In addition, the use of herbicides to prevent weed growth during the planting of young *Eucalyptus* forests inevitably affected the growth of trees, time wasted in replanting, and increased input cost.

Transgenic technology is a powerful tool to accelerate the genetic improvement of trees and solve the problems that most forest trees are facing, such as improvement in productivity, wood quality, and stress resistance (Chang et al., 2018), especially pest and herbicide intolerance in *Eucalyptus*. Although *Eucalyptus* is recalcitrant to genetic transformation, some attempts were made to introduce functional genes into *Eucalyptus* to obtain desirable phenotypes, which made it possible to cultivate new varieties by transgenic technology (Harcourt et al., 2000; Valério et al., 2003; Dibax et al., 2010; Navarro et al., 2011; Matsunaga et al., 2012; Ouyang et al., 2012; Yu et al., 2013; Aggarwal et al., 2015; Ouyang and Li, 2016; Thanananta et al., 2018). A stable and efficient regeneration system is a prerequisite for obtaining transgenic plants with ideal traits (Song et al., 2019). Many efforts have been made to establish regeneration systems by modulating concentrations of exogenous auxin and cytokinin (Hajari et al., 2006; Girijashankar, 2012; Shwe and Leung, 2020). Thidiazuron, as an important cytokinin, has also been widely used in *Eucalyptus* regeneration (Cid et al., 1999; Shabannejad Mamaghani et al., 2009; de França Bettencourt et al., 2020). Recently, *N*-phenyl-*N’*-[6-(2-chlorobenzothiazol)-yl] urea (PBU) was attempted to promote regeneration in *Eucalyptus* (Ouyang and Li, 2016). However, the regeneration efficiencies were still unstable and varied greatly among different genotypes.

*Agrobacterium tumefaciens*-mediated transformation was the most commonly utilized method in plants. This transformation protocol was also applied for various *Eucalyptus* species (Prakash and Gurumurthi, 2009; Aggarwal et al., 2011; de Alcantara et al., 2011; Silva et al., 2011; de França Bettencourt et al., 2020). The transformation efficiencies were influenced by a variety of factors including *Agrobacterium* strain, bacterial concentration, infection time, and precultivation time. Strains EHA105, LBA4404, and GV3101 were often used for transformation and showed higher efficiency (Aggarwal et al., 2011; Wang et al., 2011; Wang et al., 2022). Meanwhile, precultivation was also an important factor and the duration of preculture depended on the species of *Eucalyptus* and type of explants (Moralejo et al., 1998; de Alcantara et al., 2011). Furthermore, exogenous acetosyringone (AS) could improve transformation efficiency in *Eucalyptus* (de Alcantara et al., 2011; Silva et al., 2011). In *E. urophylla* × *E. tereticornis*, 50 μM AS in adventitious bud inducing medium noticeably improved the frequency of transformation by 8.8% (Wang et al., 2022). However, the optimal conditions are diverse in different genotypes, which means these factors should be tested in a specific genotype.

Some varieties have been successfully transformed by adjusting the phytohormone ratio, especially the use of specific hormones, and optimizing transformation methods. Unfortunately, most protocols use cotyledons or hypocotyls derived from seedlings with characteristics different from explants, which are not suitable for further transgenic breeding and gene function analysis because of gene recombination (Pena and Seguin, 2001). Clonal materials seem to be the more suitable explants for transformation because of the same genetic background and relatively stable phenotype. Although genetic transformation systems were constructed for some *Eucalyptus*, there were still some superior clones that were widely cultivated that are recalcitrant to regeneration for unknown reasons. Moreover, the transformation efficiencies, which were dependent on many factors, were genotype specific and notoriously variable. It is necessary to develop an efficient genetic transformation system by using clonal materials as explants for a specific genotype.

DH32-29, a hybrid of *E. urophylla* and *E. grandis* that was cultivated by the Dongmen forest farm in Guangxi, China, is the most widely planted superior clone in southern China because of its desirable traits, such as fast growth, high yield, short rotation period, straight trunk, high quality wood, strong resistance and wide adaptability. However, this clone has not overcome the difficulty of regeneration, which blocks the establishment of a genetic transformation system. To solve the difficulties in regenerating *E. urophylla* × *E. grandis* DH32-29 and establishing a genetic transformation system, various plant growth regulators (PGRs) and methods were adjusted using leaves obtained from micropropagated plantlets as explants. An *Agrobacterium*-mediated genetic transformation system was optimized, and transgenic plants were obtained, which made foreign genes stably integrated into the plant genome. This study will pave the way for functional analysis of genes related to superior traits and the breeding of transgenic and gene-edited high-quality varieties in the foreseeable future.

## 2 Materials and methods

### 2.1 Plant material

Shoots were cut from *E. urophylla × E. grandis* clone DH32-29 and were transferred to modified Murashige and Skoog (Murashige and Skoog, 1962) medium supplemented with 0.10 mg·L^−1^ α-naphthaleneacetic acid (NAA) and 0.50 mg·L^−1^ 6-benzyladenine (6-BA) (shoot-inducing medium, SIM) after sterilization to obtain tissue culture plantlets. The medium was replaced every 20 days to propagate the sterile plantlets.

Plantlets were cut and transferred to 1/2 MS medium supplemented with 0.10 mg·L^−1^ NAA [root-inducing medium (RIM)] to induce adventitious roots. Then, plantlets with roots were transferred to SIM and cultured in darkness for 7 days and in weak light for 14 days to gain elongated stem internodes. Stem internodes from plantlets and leaves from micropropagation plantlets were used as explants.

All media contained 30 g·L^−1^ sucrose and 7 g·L^−1^ agar, and the pH of all media was adjusted to 5.8 before autoclaving. All plantlets were cultured under a 16-h photoperiod (100 μmol·m^-2^·s^-1^) at 25 ± 2°C.

### 2.2 Establishment of an adventitious bud regeneration system

To obtain a higher frequency of adventitious bud induction, the combinations of PGRs supplemented in callus-inducing medium [CIM; woody plant medium (WPM) basal medium supplemented with different PGRs] were optimized. Stem internodes from rooted plantlets were cultured in CIM supplemented with α-naphthaleneacetic acid (NAA, 0.02–0.60 mg·L^−1^), forchlorfenuron (CPPU, 0.06–0.72 mg·L^−1^), and thidiazuron (TDZ, 0.06–0.24 mg·L^−1^) (**Supplementary Table 1**) for 15 days in darkness. Then, the stem internodes were transferred to SIM to induce adventitious buds. Based on the best combination of PGRs, leaves obtained from micropropagation plantlets were used as explants to explore the effects of callus induction time (5, 10, 15, and 20 days) on adventitious bud regeneration. The duration of subculture (15, 20, and 25 days) and different volumes of added coconut milk in CIM were explored to improve the frequency of adventitious bud induction. Then, individual buds of 3 cm were cut off and transferred to RIM to induce adventitious roots.

The growth parameters were observed daily, and the frequencies of callus and adventitious bud induction were calculated to determine the best regeneration protocol after 2 months of *in vitro* culture. The size of the callus was also calculated. Each treatment involved 120 explants and was performed in at least three replicates.

### 2.3 Determination of the critical concentration of antibiotics

Leaves were successively cultured in CIM and SIM supplemented with different concentrations and types of antibiotics, including cefotaxime (Cef) and timentin (Tmt). Buds were inoculated in RIM with the same treatments to effectively eliminate redundant *Agrobacterium* without affecting regeneration. Different concentrations of kanamycin (Kan, 0–75 mg·L^−1^) or hygromycin (Hyg, 0–5 mg·L^−1^) were supplemented in CIM, SIM, and RIM to determine the critical concentration of antibiotics and prevent untransformed plants from escaping. Fresh media were replaced every 15 days to avoid antibiotic failure. The growth parameters were observed daily. The frequencies of callus and adventitious bud induction and the size of callus were calculated after leaves were cultured for 8 weeks. The frequency of adventitious root induction and the length of roots were calculated after 4 weeks. Each treatment involved 120 explants and was performed in at least three replicates.

### 2.4 Plasmid and *Agrobacterium* strain culture

*A. tumefaciens* strains GV3101, LBA4404, EHA105, and AGL1 were used for the genetic transformation. The binary vector pBI121 contains a *neomycin phosphotransferase Ⅱ* (*nptⅡ*) selection marker gene and a *β-glucuronidase* (*uidA*) reporter gene. A single colony of *Agrobacterium* strain that contained pBI121 was cultured in 5 ml of liquid Luria–Bertani (LB) medium with 50 mg·L^−1^ Kan and 20 mg·L^−1^ rifampicin (Rif) for 48 h at 28°C with shaking at 220 rpm. A 1-ml cloudy culture was cultivated in 30 ml of liquid LB medium with the same antibiotics at 28°C until the OD_600_ was approximately 0.5. The bacterial cells were collected by centrifugation for 10 min at 4,000 rpm and 4°C, and the precipitated cells were resuspended in 30 ml of CIM liquid medium without antibiotics.

### 2.5 Optimization of the genetic transformation system

Six factors affecting the efficiency of genetic transformation were successively optimized, including *Agrobacterium* strain, concentration, duration of infection, duration of cocultivation, duration of precultivation, and concentration of the additive AS. Leaves were cut from micropropagation plantlets that were subcultured for 20–25 days, and the lower half of leaves with petioles were adopted as explants. After 0–9 days of preculture in CIM in darkness, leaves were immersed in cell suspensions of different densities (OD_600_ = 0.1, 0.3, 0.5, 0.7, and 0.9) and with different AS concentrations (0, 10, 50, 100, and 200 μM) for varying periods of time (15, 30, 60, 120, and 240 min). Subsequently, explants were placed in CIM without antibiotics and cocultured for different hours (24, 28, and 72 h) at 25 ± 2°C in darkness. After cocultivation, leaves were washed with sterile water and blotted with sterile filter paper to decontaminate *Agrobacteria*. The inoculated leaves were then successively transferred to CIM supplemented with the aforementioned selected antibiotics. After 7 days, β-glucuronidase (GUS) staining was performed.

### 2.6 Screening of resistant plants

Based on the optimal genetic transformation system, leaves were transferred to CIM containing antibiotics and cultured for 15 days to induce callus formation. Then, the leaves were replaced by SIM supplemented with antibiotics to obtain resistant adventitious buds. The fresh SIM was replaced every 15 days until resistant adventitious buds regenerated. Regenerated adventitious buds with 2 cm were excised and transferred to RIM supplemented with 200 mg·L^−1^ Cef and 75 mg·L^−1^ Kan for adventitious root induction. Each treatment involved 120 explants and was performed in at least three replicates.

### 2.7 GUS histochemical assay

Leaves transformed with *Agrobacterium* and putatively transgenic plants were detected by GUS histochemical staining as previously described (Jefferson et al., 1987). The plant materials were immersed in a reagent with 2 mM 5-bromo-4-chloro-3-indolyl-β-D-glucuronide (X-Gluc), 0.5 mM potassium ferrocyanide, 0.5 mM potassium ferricyanide, 0.1 M Na-phosphate buffer (pH 7.0), and 0.1% (v/v) Triton X-100 and were incubated at 37°C for 12 h. Then, the staining solution was replaced by 70% ethanol, and the plant materials were washed four to six times to completely remove the chlorophyll. Owing to most adventitious buds that originated from petioles, the frequency of GUS staining of petioles and leaves was calculated separately to accurately present transformation efficiency.

### 2.8 Molecular analysis of transgenic plants

Genomic DNA was isolated from the leaves of wild-type and putatively transgenic plants using a modified cetyltrimethylammonium bromide (CTAB) method. The *nptⅡ* gene and *uidA* gene were amplified by polymerase chain reaction (PCR) amplification using specific primers (*nptⅡ*-F: TTGAACAAGATGGATTGCACGCA, *nptⅡ*-R: GAGCGGCGATACCGTAAAGCA, *uidA*-F: ACTAGCAAGCGCACTTACAGG, *uidA*-R: TCCATACCTGTTCACCGACGAC). The 2 × Taq PCR StarMix (Genstar, Fuzhou, Cangshan, China) was used according to the manufacturer’s instructions. The PCR program was as follows: initial denaturing at 94°C for 5 min, followed by 35 cycles at 94°C for 30 s, 58°C (*nptII*) or 56°C (*uidA*) for 30 s, 72°C for 45 s (*nptII*) or 30 s (*uidA*), and a final extension for 7 min at 72°C.

### 2.9 Statistical analysis

All experiments including adventitious bud induction, determination of antibiotic concentration, and optimization of genetic transformation were performed in triplicate in different periods and each replicate contained at least 120 explants (more than six dishes and 20 explants per dish). The number of explants that regenerated calluses and adventitious buds and stained blue was recorded to calculate the frequency of adventitious bud regeneration and genetic transformation. The size of the callus was calculated as follows: explant with no callus was denoted as 0, leaf with callus but little enlargement was denoted as 1, leaf with callus and enlargement 1.5 times the original volume was denoted as 2, leaf with callus and enlargement 2 times the original volume was denoted as 3, and leaf with callus and enlargement 3 times the original volume was denoted as 4. SPSS 26 was used for all data analyses. Data were transformed by the following formula and further assessed if a set of data coincided with normal contributions (**Supplementary File 1**). Then, the transformed data were analyzed using one-way analysis of variance (ANOVA) followed by Duncan’s multiple range test. The different letters in the graphs indicate significant differences among treatments (*p*< 0.05).


X'=ASIN(SQRT(X))

## 3 Results

### 3.1 Efficient adventitious bud regeneration of *E. urophylla* × *E. grandis* DH32-29

**Figure 1:**
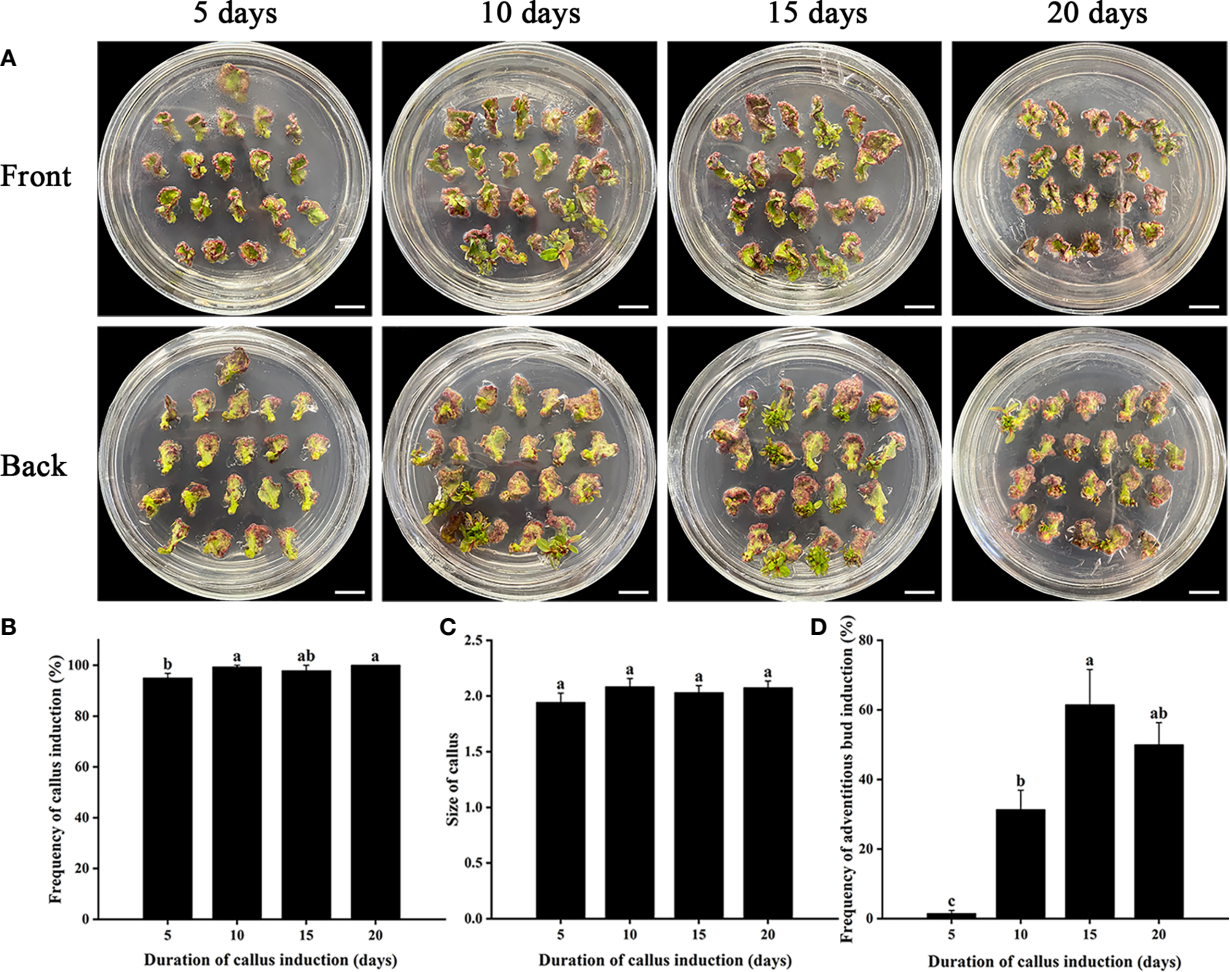
Effects of different durations of callus induction on adventitious bud induction of *E. urophylla* × *E. grandis* DH32-29. **(A)** Explants are induced to form calli for 5, 10, 15, and 20 days. Effects of different durations of callus induction on **(B)** the frequency of callus induction, **(C)** callus size, and **(D)** the frequency of adventitious bud induction. Scale bar: 1 cm. Different letters indicate significant differences among treatments using Duncan’s multiple range test at *p*< 0.05.

To establish an efficient adventitious bud regeneration system of *E. urophylla* × *E. grandis* DH32-29, different hormone combinations containing PGRs (including NAA, CPPU, and TDZ) were adopted based on the previous studies and preliminary experiments (data not shown) (**Supplementary Table 1**). However, the adventitious bud induction rate was low regardless of which hormone combination treatment with stem internodes as explants (**Supplementary Table 1**). The highest adventitious bud regeneration rate was obtained by stem internodes when 0.02 mg·L^−1^ NAA was combined with 0.24 mg·L^−1^ CPPU (**Supplementary Table 1**). The frequency of adventitious bud induction increased from 17.2% to 61.4% when stems were replaced by leaves and were cultured in liquid CIM supplemented with 0.02 mg·L^−1^ NAA and 0.24 mg·L^−1^ CPPU for 15 days (**Figure 1**), indicating that leaves were more suitable as explants for further genetic transformation. The induction of calli is very important for adventitious bud regeneration. A duration of callus induction that was too short (5–10 days) or too long (20 days) was not advantageous for adventitious bud induction (**Figure 1**). The highest frequency of adventitious bud induction (61.4%) was obtained when leaves were induced to form calluses for 15 days (**Figure 1**), and the number of adventitious buds regenerated from each explant was more than 5.

The effects of the condition of leaves on adventitious bud regeneration were tested. Subculture times (15, 20, and 25 days) of plantlets had no influence on adventitious bud regeneration (**Supplementary Figure 1**). Leaves from plantlets subcultured for 20-25 days were used as explants because of the proper size for operation. Furthermore, different proportions of coconut milk and WPM were used to improve the frequency of adventitious bud induction. However, the color of the explants changed to yellow-green with the increase in the volume of added coconut milk, and the frequency of adventitious bud induction gradually decreased (**Supplementary Figure 2**). When the WPM medium was replaced by 100% coconut milk, the adventitious bud induction rate decreased to 21.5% (**Supplementary Figure 2D**). Thus, coconut milk was not required in the medium to induce callus formation.

**Figure 2:**
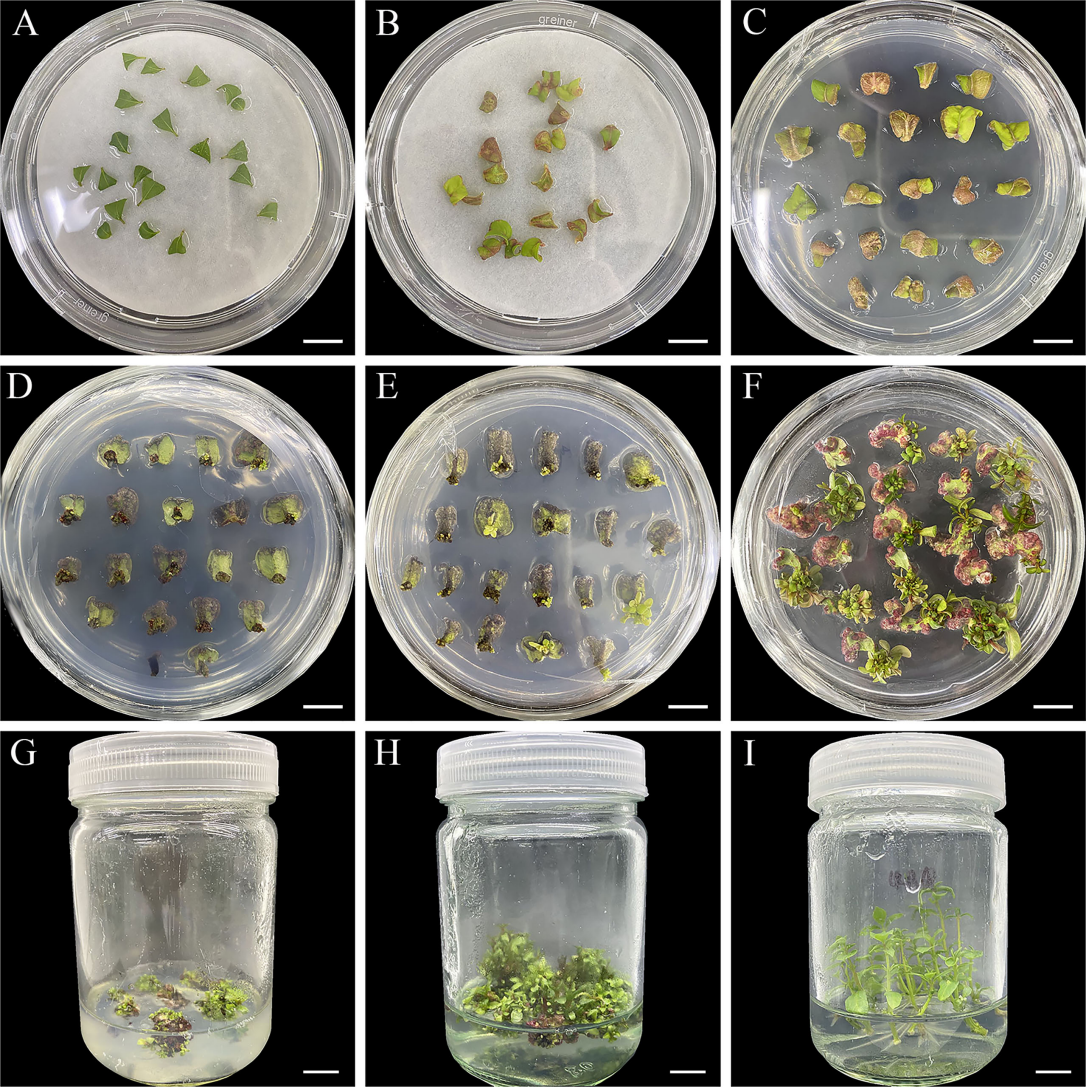
Adventitious bud regeneration from leaves of *E. urophylla* × *E. grandis* DH32-29. The explants were cultured in the medium for **(A)** 0, **(B)** 3, **(C)** 15, **(D)** 30, **(E)** 45, and **(F)** 60 days. Regenerated buds are propagated **(G, H)** and rooted **(I)**. Scale bar: 1 cm.

The complete regeneration process of *E. urophylla* × *E. grandis* DH32-29 is shown in **Figure 2**. Leaves from plantlets subcultured for 20–25 days were cut off, and the upper part was removed. Then, they were cultured in CIM (liquid WPM basal medium supplemented with 0.02 mg·L^−1^ NAA and 0.24 mg·L^−1^ CPPU) for 15 days to induce calluses (**Figure 2A**). After 3 days, the leaves began to swell and became discolored, and calluses were forming (**Figure 2B**). After 15 days, leaves were transferred to SIM and continued to expand, and calluses were further developing (**Figure 2C**). Green bud spots appeared on the petioles of leaves after 1 month of *in vitro* culture (**Figure 2D**). The regenerated adventitious buds gradually grew and elongated (**Figures 2E–H**), and they were cut into 1-cm pieces and transferred to RIM when the length of regenerated adventitious buds was more than 3 cm (**Figure 2I**). Taken together, the regeneration of *E. urophylla* × *E. grandis* DH32-29 from leaves to form a whole plantlet was accomplished in approximately 2 months. The highest adventitious bud rate was 76.5% (**Supplementary Figure 2D**) after optimization.

### 3.2 Determination of the critical concentration of antibiotics

**Figure 3:**
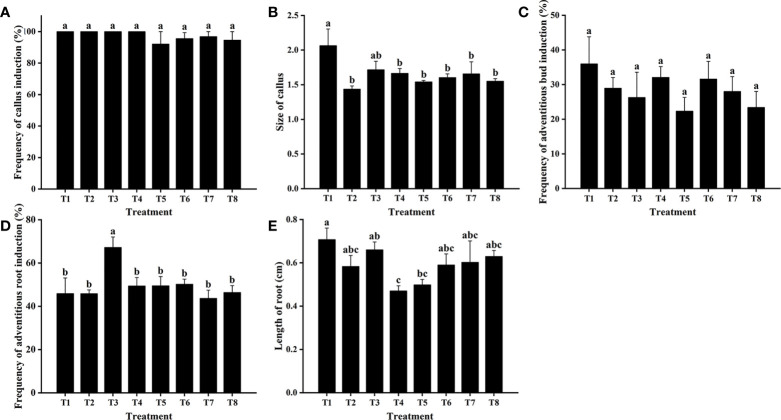
Effects of cefotaxime (Cef) and timentin (Tmt) on regeneration of *E. urophylla* × *E. grandis* DH32-29. Effects of Cef and Tmt on **(A)** the frequency of callus induction, **(B)** callus size, **(C)** frequency of adventitious bud induction, **(D)** frequency of adventitious root induction, and **(E)** length of roots. Combinations of antibiotics: T1: 0 mg·L^−1^ Cef and 0 mg·L^−1^ timentin (Tmt), T2: 100 mg·L^−1^ Cef and 0 mg·L^−1^ Tmt, T3: 200 mg·L^−1^ Cef and 0 mg·L^−1^ Tmt, T4: 300 mg·L^−1^ Cef and 0 mg·L^−1^ Tmt, T5: 400 mg·L^−1^ Cef and 0 mg·L^−1^ Tmt, T6: 100 mg·L^−1^ Cef and 100 mg·L^−1^ Tmt, T7: 100 mg·L^−1^ Cef and 50 mg·L^−1^ Tmt, T8: 200 mg·L^−1^ Cef and 50 mg·L^−1^ Tmt. Different letters indicate significant differences among treatments using Duncan’s multiple range test at *p*< 0.05.

**Figure 4:**
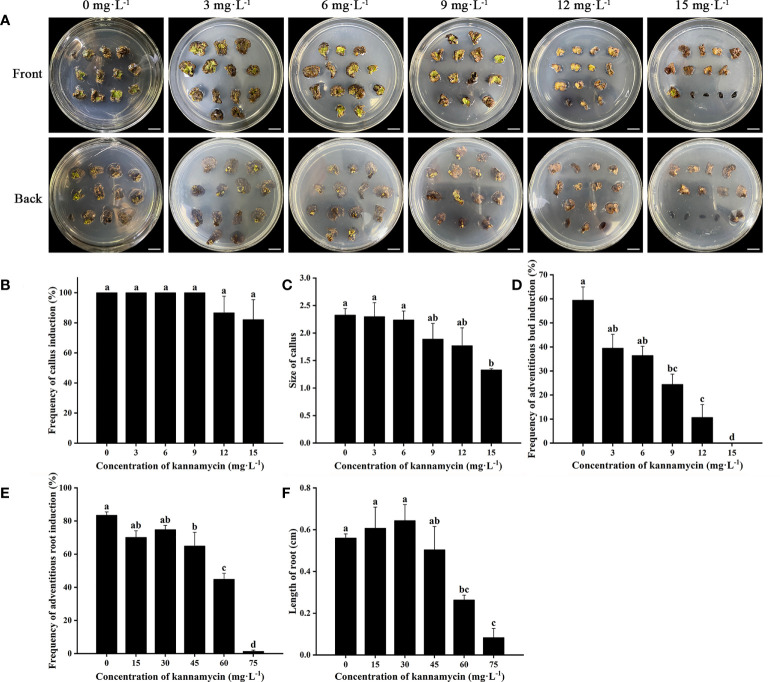
Effects of kanamycin on regeneration of *E. urophylla* × *E. grandis* DH32-29. **(A)** Explants are induced to regenerate adventitious buds on medium supplemented with 0, 3, 6, 9, 12, and 15 mg·L^−1^ kanamycin. Effects of kanamycin on **(B)** the frequency of callus induction, **(C)** callus size, **(D)** the frequency of adventitious bud induction, **(E)** the frequency of adventitious root induction, and **(F)** the length of roots. Scale bar: 1 cm. Different letters indicate significant differences among treatments using Duncan’s multiple range test at *p*< 0.05.

**Figure 5:**
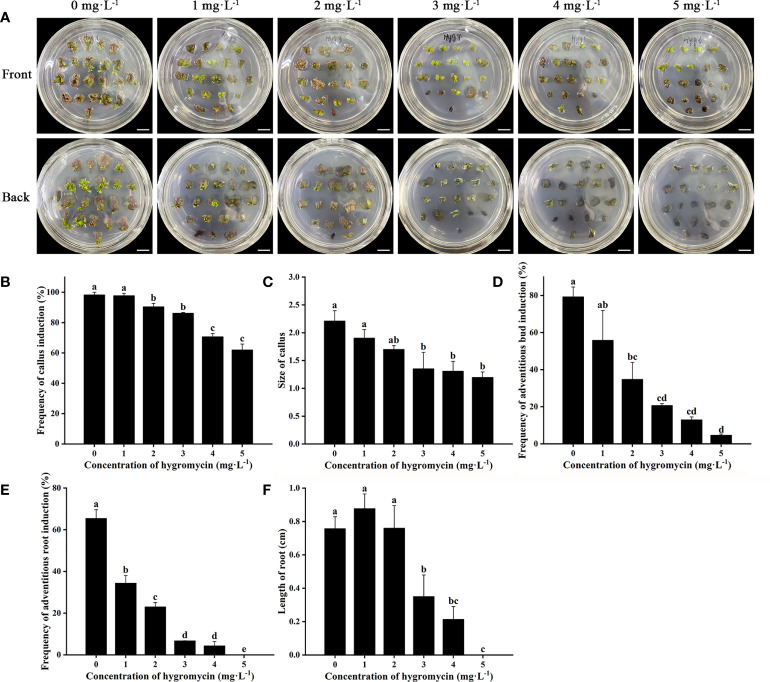
Effects of hygromycin on regeneration of *E. urophylla* × *E. grandis* DH32-29. **(A)** Explants are induced to regenerate adventitious buds on medium supplemented with 0, 1, 2, 3, 4, and 5 mg·L^−1^ hygromycin. Effects of hygromycin on **(B)** the frequency of callus induction, **(C)** callus size, **(D)** the frequency of adventitious bud induction, **(E)** the frequency of adventitious root induction, and **(F)** the length of root. Scale bar: 1 cm. Different letters indicate significant differences among treatments using Duncan’s multiple range test at *p*< 0.05.

Cefotaxime and timentin are usually used to inhibit the excessive proliferation of *Agrobacterium*. The induction percentage of calli and adventitious buds was not significantly affected by supplementation with Cef and Tmt (**Figures 3A–C**). Calli were also found to decrease in size, which would be helpful for further antibiotic selection. The combination of 100 mg·L^−1^ Cef and 100 mg·L^−1^ Tmt (T6) was chosen for selecting transgenic adventitious buds because of the preferable inhibition effect. A total of 200 mg·L^−1^ Cef (T3) was supplemented in RIM for the higher adventitious root induction rate (**Figures 3D, E**). Leaves were cultured in CIM and SIM supplemented with a series of concentrations of Kan or Hyg to determine the critical concentration for selection. The regeneration of adventitious buds was totally inhibited when Kan was applied at a concentration of 15 mg·L^−1^ (**Figure 4**). In contrast, the formation of adventitious roots was nearly inhibited when the concentration of Kan reached 75 mg·L^−1^ in RIM (**Figures 4E, F**). Hence, 15 mg·L^−1^ and 75 mg·L^−1^ Kan were used to screen transgenic adventitious buds and roots, respectively. Interestingly, both adventitious bud regeneration and root formation were highly sensitive to Hyg and 5 mg·L^−1^ Hyg almost completely inhibited the regeneration of adventitious buds and root formation (**Figure 5**). Finally, 5 mg·L^−1^ Hyg was determined for the selection of transgenic plantlets with the hygromycin resistance gene.

### 3.3 Optimization of an *Agrobacterium*-mediated transformation system for *E. urophylla* × *E. grandis* DH32-29

**Figure 6:**
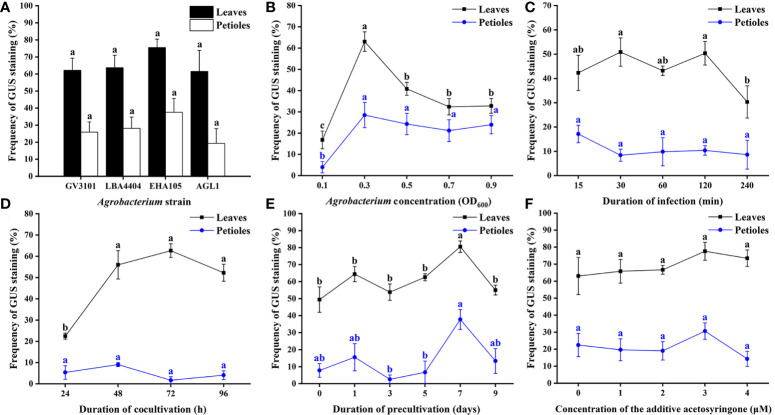
Optimization of *Agrobacterium*-mediated genetic transformation of *E. urophylla* × *E. grandis* DH32-29. Effect of **(A)** *Agrobacterium* strain, **(B)** *Agrobacterium* concentration, **(C)** duration of infection, **(D)** duration of cocultivation, **(E)** duration of precultivation, and **(F)** concentration of the additive acetosyringone on the transformation rate. Different letters indicate significant differences among treatments using Duncan’s multiple range test at *p*< 0.05.

The transformation efficiency showed no obvious difference in the GV3101, EHA105, LBA4404, and AGL1 strains (**Figure 6A**). EHA105 was chosen as the bacterial strain for the following optimization because most transgenic plants were infected with EHA105. The highest transformation rate of leaves reached 63.1% when they were infected with the bacterial suspension of 0.3 (**Figure 6B**). There was no significant difference in the transformation efficiency of leaves when they were infected for 30 or 120 min (**Figure 6C**). However, the longer infection time caused damage to explants, resulting in browning of leaves in the subsequent selective culture. Thus, 30 min was chosen for subsequent experiments. Cocultivation time was also performed to promote the transformation. The result showed that cocultivation for 72 h reached 62.6% transformation efficiency, which benefitted the transfer and integration of T-DNA and avoided the overgrowth of *Agrobacterium* (**Figure 6D**). Moreover, the transformation efficiency was significantly increased when leaves were precultured for 7 days (**Figure 6E**). Additionally, AS supplemented in bacterial suspension was not observed to significantly improve the frequency of genetic transformation in this study (**Figure 6F**).

### 3.4 Verification of putative transformants by GUS staining and PCR

**Figure 7:**
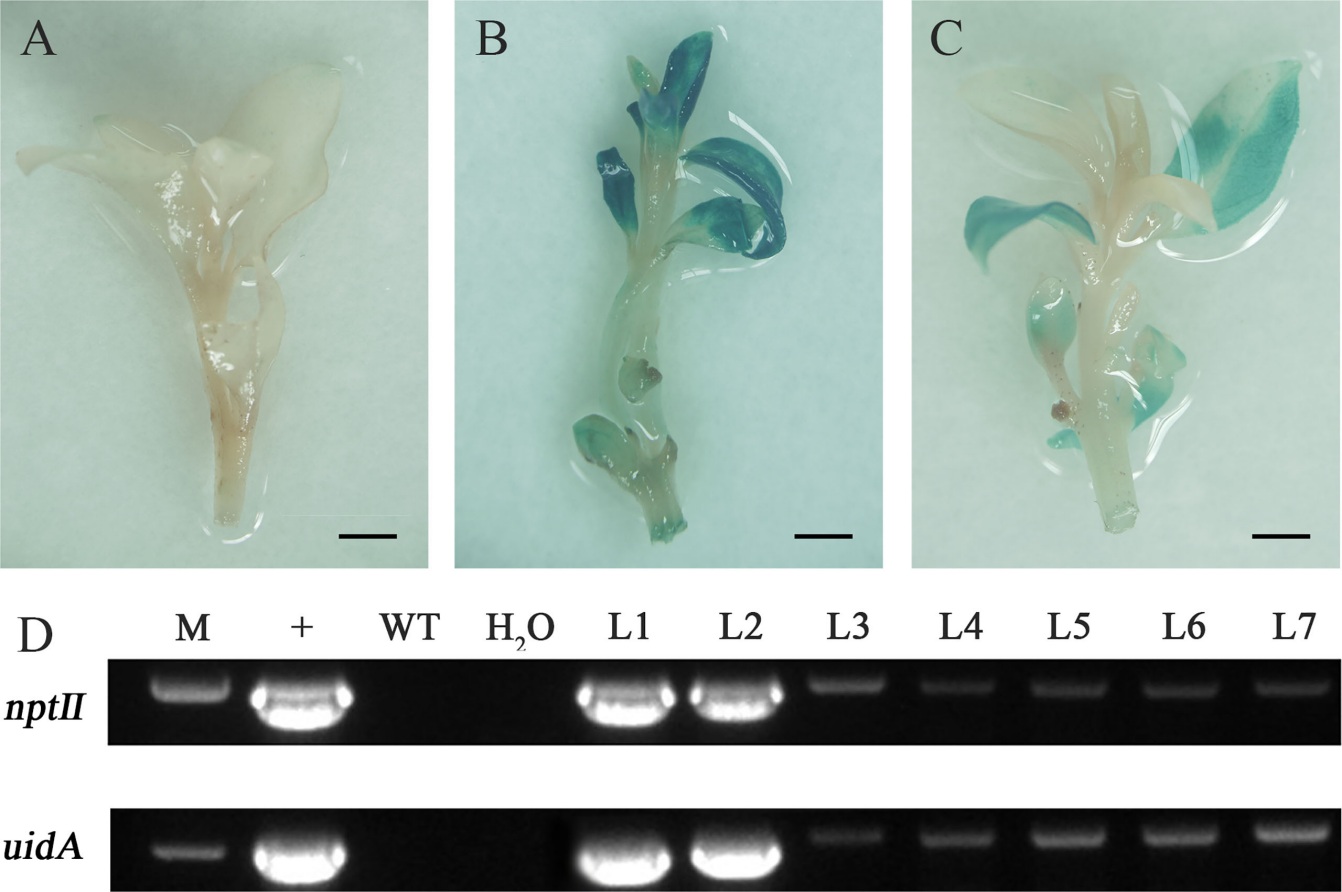
Molecular detection and β-glucuronidase (GUS) expression in transgenic plants. **(A)** Negative control. **(B)** and **(C)** GUS staining was observed in transgenic plants. **(D)** PCR amplification of the *nptⅡ* gene and *uidA* gene in pBI121: (M) marker, (+) plasmid, (WT) untransformed wild-type, (H_2_O) water control, and (L1-L7) transgenic lines. Scale bar: 0.25 cm.

The regenerated adventitious buds showing resistance to Kan were analyzed by GUS staining and PCR amplification to detect the integration of the *nptⅡ* and *uidA* genes. Finally, seven regenerated plants were verified as positive transformants from 360 infected leaves in three experiments, with a 1.9% transformation rate (**Figure 7**). The negative control, in which adventitious buds were not infected, showed that GUS dyeing failed, and no target band was amplified in the negative control. Some resistant adventitious buds were chimeric blue, which might be caused by uneven staining or different expression levels of the *uidA* gene at different locations.

### 3.5 The complete genetic transformation protocol of *E. urophylla* × *E. grandis* DH32-29

**Figure 8:**
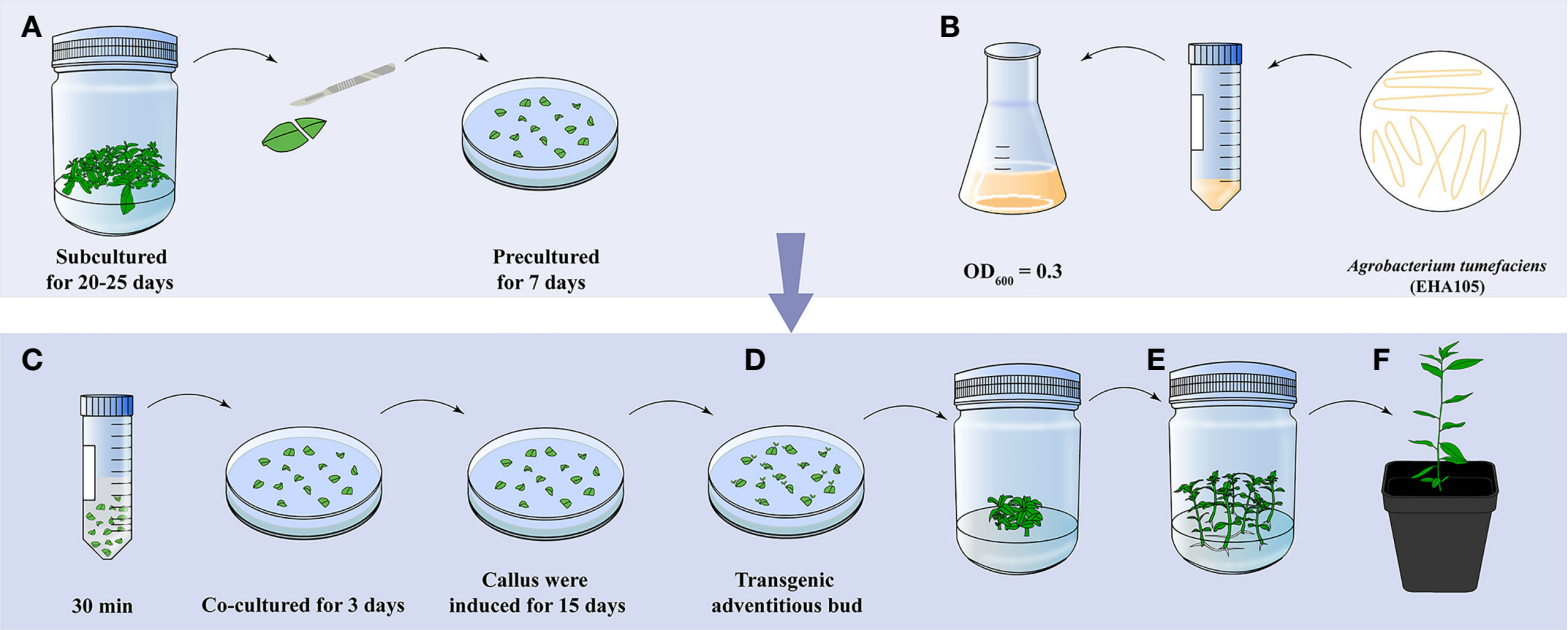
*Agrobacterium*-mediated *E. urophylla* × *E. grandis* DH32-29 leaf transformation workflow. **(A)** Leaves were cut from plantlets that were subcultured for 20–25 days and precultured in callus-inducing medium for 7 days. **(B)** *Agrobacterium* EHA105 was cultured to 0.3 (OD_600_) and **(C)** infected leaves for 30 min. Leaves were cocultured for 72 h and transferred to callus-inducing medium to induce calluses. **(D)** Transgenic adventitious buds were generated when explants were cultured in shoot-inducing medium. **(E)** Transgenic buds were propagated, induced roots, and **(F)** planted in the soil.

The whole workflow of genetic transformation in *E. urophylla* × *E. grandis* DH32-29 was drawn and is shown in **Figure 8**. Leaves were cut from plantlets that were subcultured for 20–25 days and laid on CIM supplemented with 0.02 mg·L^−1^ NAA and 0.24 mg·L^−1^ CPPU. After 7 days, the explants were transferred to an *Agrobacterium* EHA105 suspension of 0.3 (OD_600_) for infiltration for 30 min. Then, the explants were moved to coculture medium and cultivated for 72 h at 25°C to transfer exogenous genes and integrate them into plant cells. Next, explants were cultured in CIM supplemented with 100 mg·L^−1^ Cef, 100 mg·L^−1^ Tmt, and 15 mg·L^−1^ Kan. Fifteen days later, explants were transferred to SIM supplemented with the same concentration of antibiotics as in CIM, and the same medium was replaced every 15 days until resistant adventitious buds gradually appeared. Putatively transgenic buds were cut and transferred to RIM supplemented with 200 mg·L^−1^ Cef and 75 mg·L^−1^ Kan to induce adventitious root formation. Finally, verified transgenic shoots were planted in the soil and grown in a greenhouse.

## 4 Discussion

Due to the huge cultivated area in China and high economic value, the establishment of a regeneration and genetic transformation system of *Eucalyptus* has received increasing attention. Although many *Eucalyptus* species have been transformed successfully in the past two decades (Ho et al., 1998; Moralejo et al., 1998; Prakash and Gurumurthi, 2009; Aggarwal et al., 2011; de Alcantara et al., 2011; Silva et al., 2011), sexual materials used as explants have resulted in differences between the mother tree and descendants, which has limited the application of transgenic plants. As the largest planted area tree species in southern China, the establishment of a stable and efficient genetic transformation system of *Eucalyptus urophylla* × *E. grandis* DH32-29 has practical value. The resulting transgenic lines with genotypes improved by genetic transformation can be used directly in afforestation and gene function analysis. Hence, it is necessary to establish an efficient adventitious bud regeneration and *Agrobacterium*-mediated genetic transformation system for *E. urophylla* × *E. grandis* DH32-29.

Many factors affect plant regeneration and PGRs play a crucial role in plant development and regeneration. Our research group successfully used TDZ to induce regeneration of a variety of eucalyptus plants. However, TDZ did not work very well on *E. urophylla* × *E. grandis* DH32-29, which also indicated that this clone is difficult to regenerate. Other cytokinin hormones, such as 6-BA, kinetin, and zeatin, could also not promote adventitious bud regeneration in many preliminary experiments. We found that the addition of CPPU observably enhanced the regeneration of adventitious buds. A previous study showed that the combination of CPPU and NAA was effective for the induction of nodule cultures in *Eucalyptus* that could regenerate shoots, and the effective concentration ranged from 0.2 to 0.5 mg·L^−1^ (Ito et al., 1996), consistent with our results that 0.24 mg·L^−1^ CPPU promoted callus formation and adventitious bud regeneration. High contents of Ca, K, and Mg were reported to be detected in CPPU-containing medium, and CPPU enhanced regeneration and adventitious bud elongation of *Jatropha curcas* because of the high mineral uptake (Singh, 2017), which might explain the accelerative effect of CPPU.

The formation of calli is very important to regenerate adventitious buds. Calluses of different ages showed different growth capacities and regeneration abilities (Cheng et al., 2009; Carsono et al., 2021), which was also shown in our study. The highest frequency of callus and adventitious bud induction of *E. urophylla* × *E. grandis* DH32-29 was obtained when leaves were used to induce calluses for 15 days. Young calluses were reported to contain more dividing cells, which were more sensitive to shoot initiation and development (Manimaran et al., 2013). In addition, liquid WPM media were used in our research instead of normal solid media, which promoted the absorption of nutrients by explants. In addition, the physiological status of explants affected the regeneration ability. Propagated plantlets with shorter days after subculture had a higher frequency of adventitious bud induction, and older aseptic plantlets had a lower regeneration rate due to the lack of nutrients or the accumulation of certain metabolites (Lv et al., 2005), which may be relevant to the expression of miR156, whose decreased expression level in old plants promotes the transcription of miR156-targeted Squamosa promoter binding protein-like (SPL) transcription factors, which affect the cytokinin signaling pathway (Zhang et al., 2015). However, plants with different durations of subculture had no significant difference in the frequency of adventitious bud induction in this research, which might be due to the comprehensive effect of multiple factors on explant status.

Suitable selection pressure allows normal growth of transgenic plants and prevents untransformed plants from escaping. Therefore, it is essential to determine the critical concentration of antibiotics to obtain transgenic plants. Previous studies have shown that great differences exist in the kanamycin tolerance of different species, which varies from 12.5 to 110 mg·L^−1^ and varies with the type of explant (Prakash and Gurumurthi, 2009; Silva et al., 2010; Guo et al., 2012; de França Bettencourt et al., 2020; Wang et al., 2022). *Eucalyptus* was more sensitive to hygromycin, and a low concentration was enough to inhibit plant regeneration (Fernando et al., 2016). In our research, no adventitious buds regenerated under 15 mg·L^−1^ Kan treatment or 5 mg·L^−1^ Hyg treatment. Vast differences existed in the response to antibiotics in adventitious buds and roots of *E. urophylla* × *E. grandis* DH32-29. Thus, different concentrations of antibiotics during genetic transformation are required for the induction of adventitious buds and roots.

The chimeric blue of GUS staining and large variation in PCR performance implied that some transgenic plants may be chimeric (**Figures 7C, D**). Although 15 mg·L^−1^ Kan was added in the process of callus and shoot induction, some untransformed cells proliferated and formed partial tissue of regenerated shoots. The phenomenon of chimera is very common in *Agrobacterium*-mediated transformation, which was probably the result that the untransformed cells could be protected through efficient detoxification of the antibiotic by the transformed cells (Chen, 2011). The increased concentration of antibiotics may reduce the frequency of chimeras, but at the cost of fewer regenerated buds. Another strategy is to induce multiple rounds of shoot regeneration, which could significantly reduce the frequency of chimeras in poplar (Ding et al., 2020).

In summary, we established and optimized an *Agrobacterium*-mediated genetic transformation system using leaves as explants in *E. urophylla* × *E. grandis* DH32-29. The frequency of genetic transformation was almost 1.9%, which made it possible to identify some key genes involved in their superior traits, such as fast growth and wide adaptability of *Eucalyptus*. Moreover, this system can be used for producing transgenic lines with modified traits, especially in improving the tolerance of insects and herbicides for further afforestation.

## Data availability statement

The original contributions presented in the study are included in the article/**Supplementary Material**. Further inquiries can be directed to the corresponding author.

## Author contributions

CF conceived this study. XW, SC, HZ, PL, and FZ performed the experiments. XW analyzed the data. XW and CF wrote the manuscript. BZ and JX revised and gave advice for the manuscript. All authors read and approved the final manuscript.

## Supplementary material

Supplementary Figure 1 Effects of the duration of explant subculture on adventitious bud induction of *E. urophylla* × *E. grandis* DH32-29. Effects of different durations of subculture on the frequency of callus induction **(A)**, callus size **(B)**, and the frequency of adventitious bud induction **(C)**. Different letters indicate significant differences among treatments using Duncan’s multiple range test at *p*< 0.05.

Supplementary Figure 2 Effects of different volumes of additive coconut milk on adventitious bud induction of *E. urophylla* × *E. grandis* DH32-29. **(A)** Explants are induced by 0%, 20%, 40%, 60%, 80%, and 100% (volume) coconut milk supplemented in callus-inducing medium. Effects of different volumes of added coconut milk on **(B)** the frequency of callus induction, **(C)** callus size, and **(D)** the frequency of adventitious bud induction. Scale bar: 1 cm. Different letters indicate significant differences among treatments using Duncan’s multiple range test at *p*< 0.05.

Supplementary File Q-Q plots of transformed data and *p*-value.
