# Supplementary figures and images for: Agrobacterium-mediated genetic transformation of the most widely cultivated superior clone Eucalyptus urophylla × E. grandis DH32-29 in Southern China
Source: Front Plant Sci. 2023 Jan 17;13:1011245. doi: 10.3389/fpls.2022.1011245 (PMC9886895; doi:10.3389/fpls.2022.1011245)

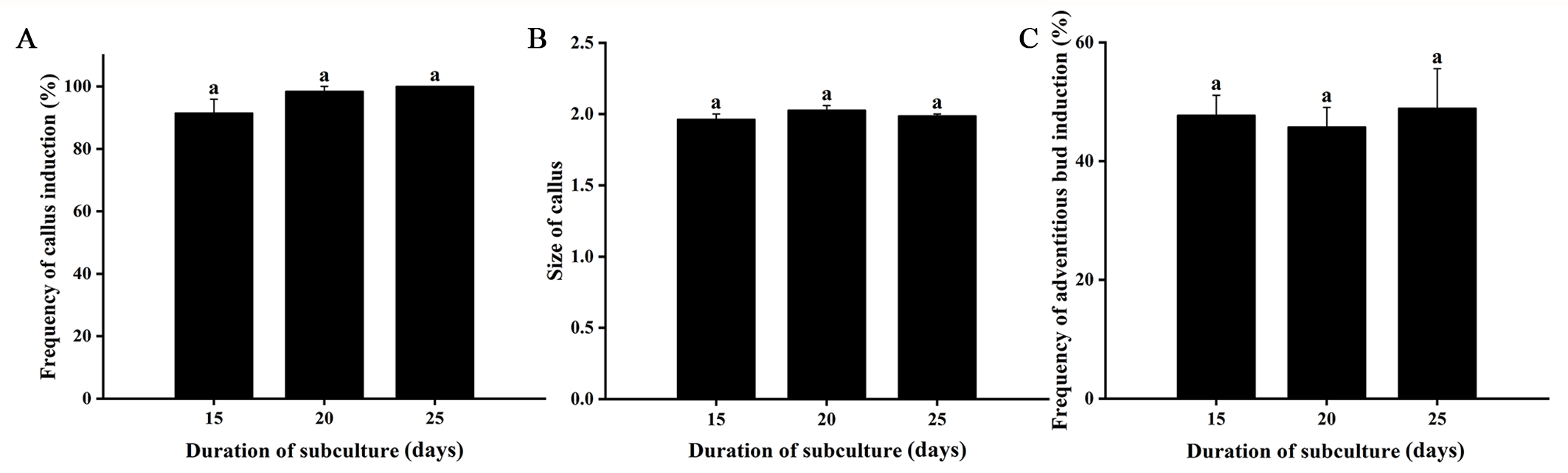

Supplement: Supplementary Figure 1 — Effects of the duration of explant subculture on adventitious bud induction of E. urophylla × E. grandis DH32-29. Effects of different durations of subculture on the frequency of callus induction (A), callus size (B), and the frequency of adventitious bud induction (C). Different letters indicate significant differences among treatments using Duncan’s multiple range test at p< 0.05. [file Image_1.tif]

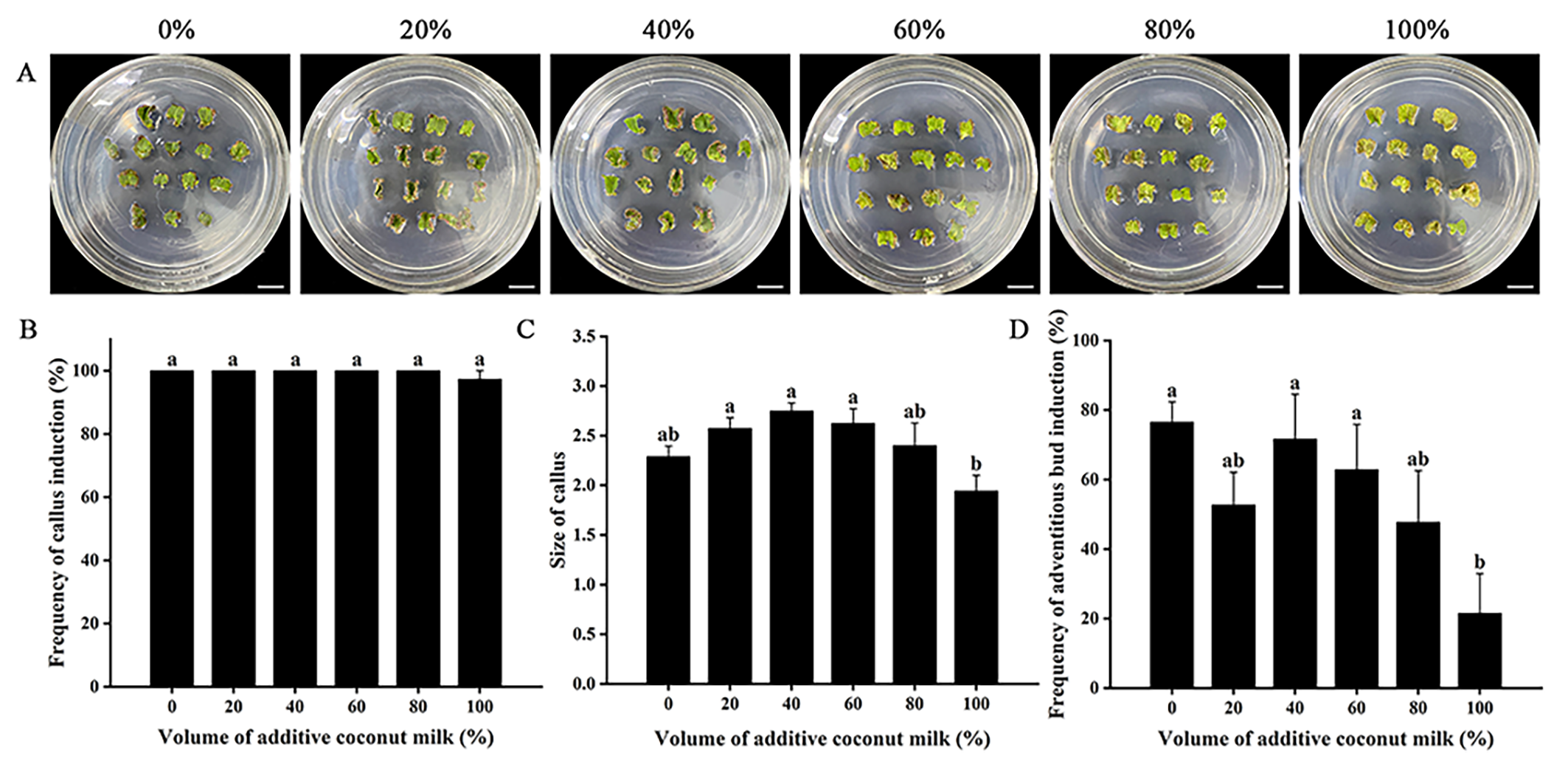

Supplement: Supplementary Figure 2 — Effects of different volumes of additive coconut milk on adventitious bud induction of E. urophylla × E. grandis DH32-29. (A) Explants are induced by 0%, 20%, 40%, 60%, 80%, and 100% (volume) coconut milk supplemented in callus-inducing medium. Effects of different volumes of added coconut milk on (B) the frequency of callus induction, (C) callus size, and (D) the frequency of adventitious bud induction. Scale bar: 1 cm. Different letters indicate significant differences among treatments using Duncan’s multiple range test at p< 0.05. [file Image_2.tif]
