# Supplementary material for: Agrobacterium-mediated genetic transformation of the most widely cultivated superior clone Eucalyptus urophylla × E. grandis DH32-29 in Southern China
Source: Front Plant Sci. 2023 Jan 17;13:1011245. doi: 10.3389/fpls.2022.1011245 (PMC9886895; doi:10.3389/fpls.2022.1011245)
Supplement: Supplementary File — Q-Q plots of transformed data and p-value. [file DataSheet_1.docx]

1. Q-Q plot of Figure 1


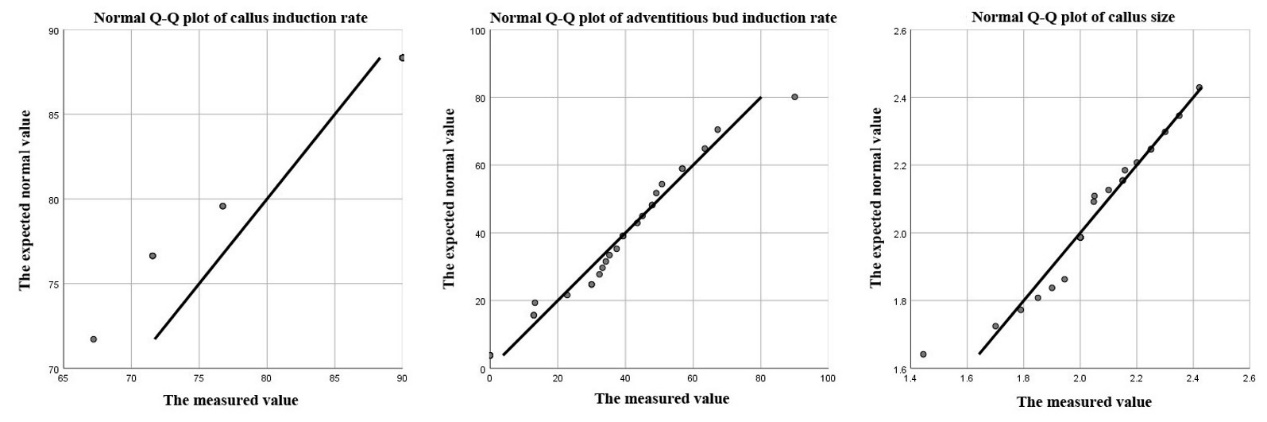


1. Q-Q plot of Figure 3


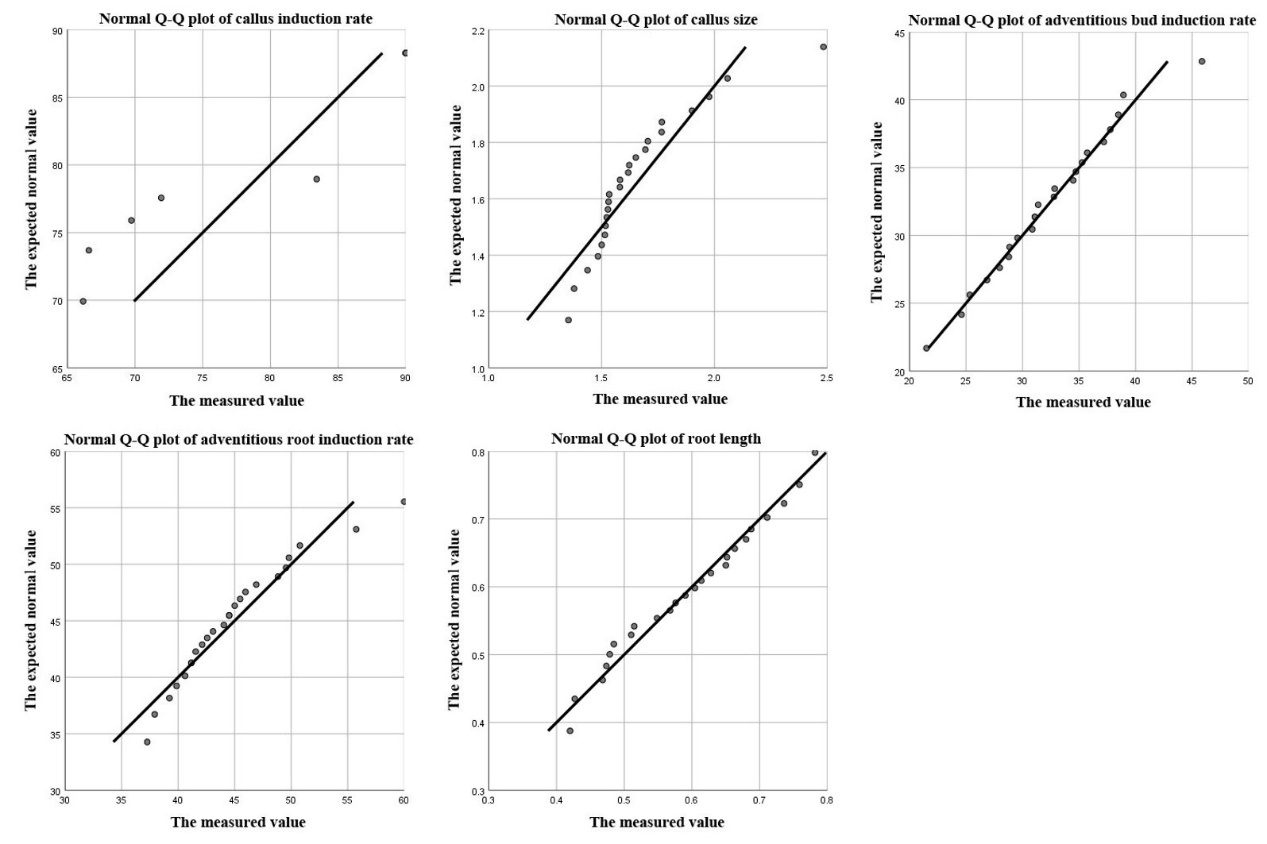


1. Q-Q plot of Figure 4


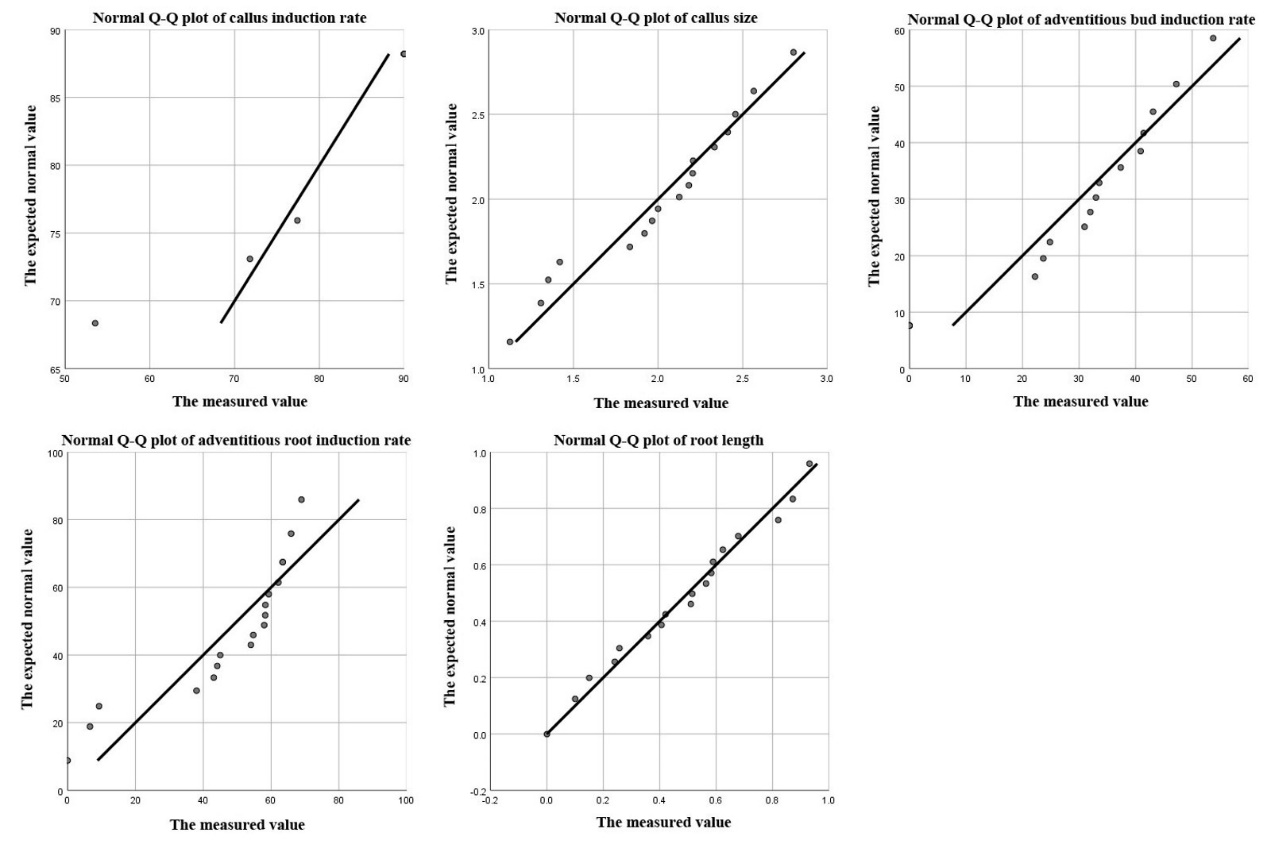


1. Q-Q plot of Figure 5


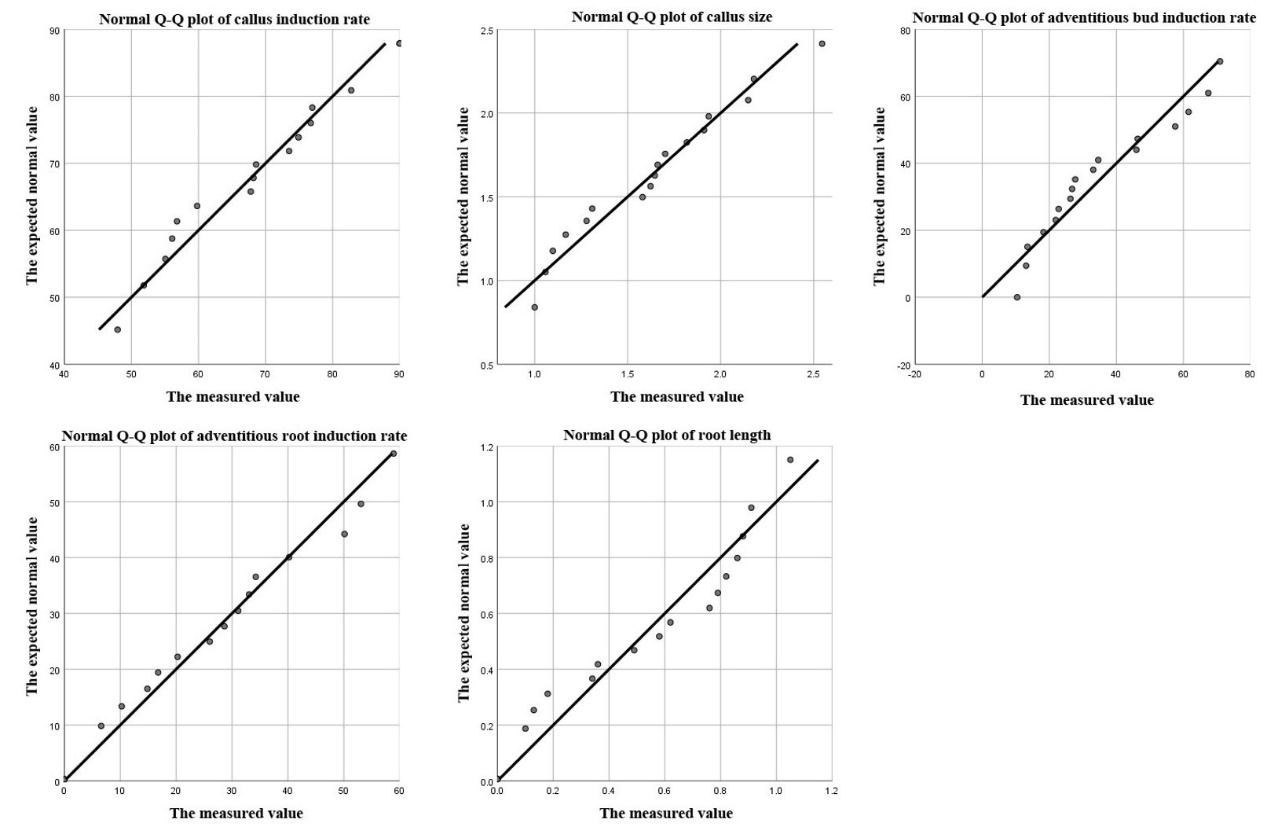


1. Q-Q plot of Figure 6


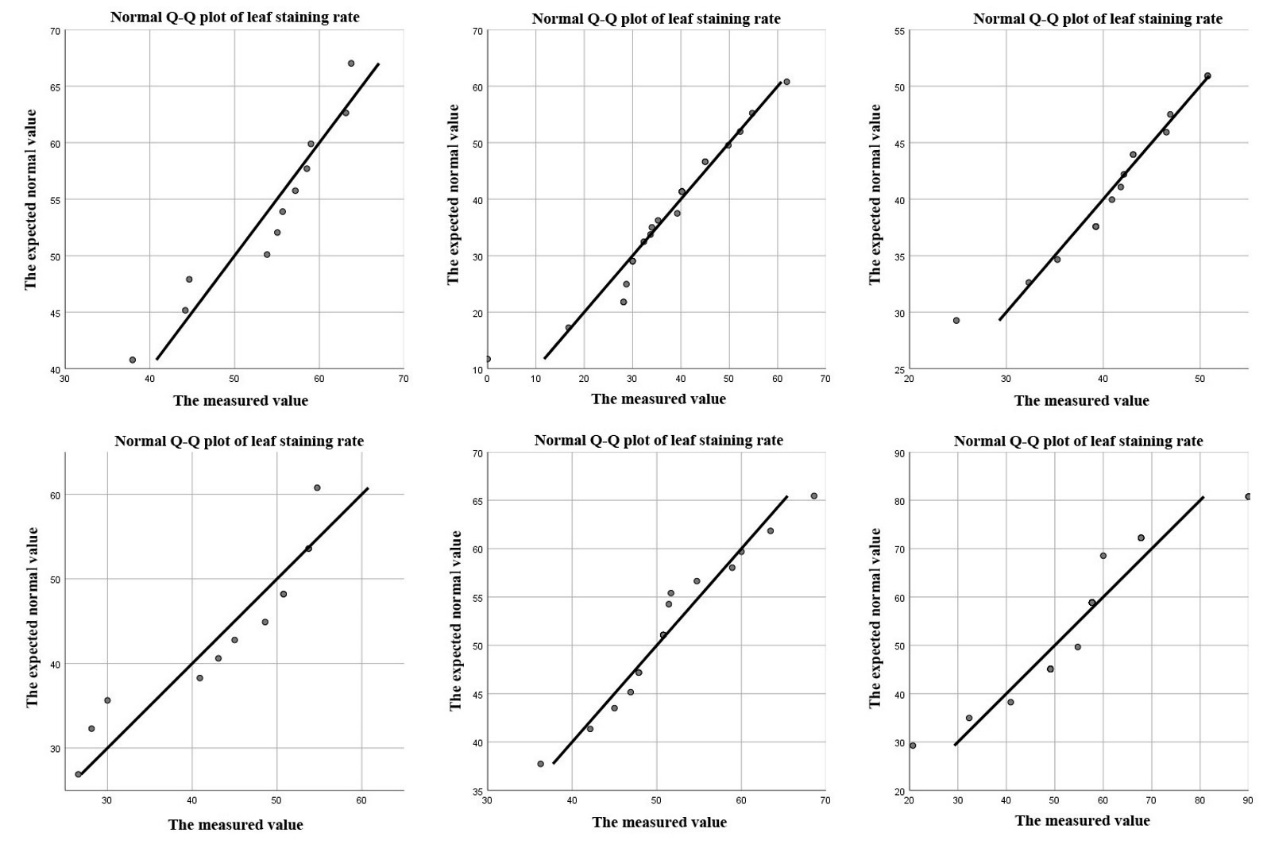


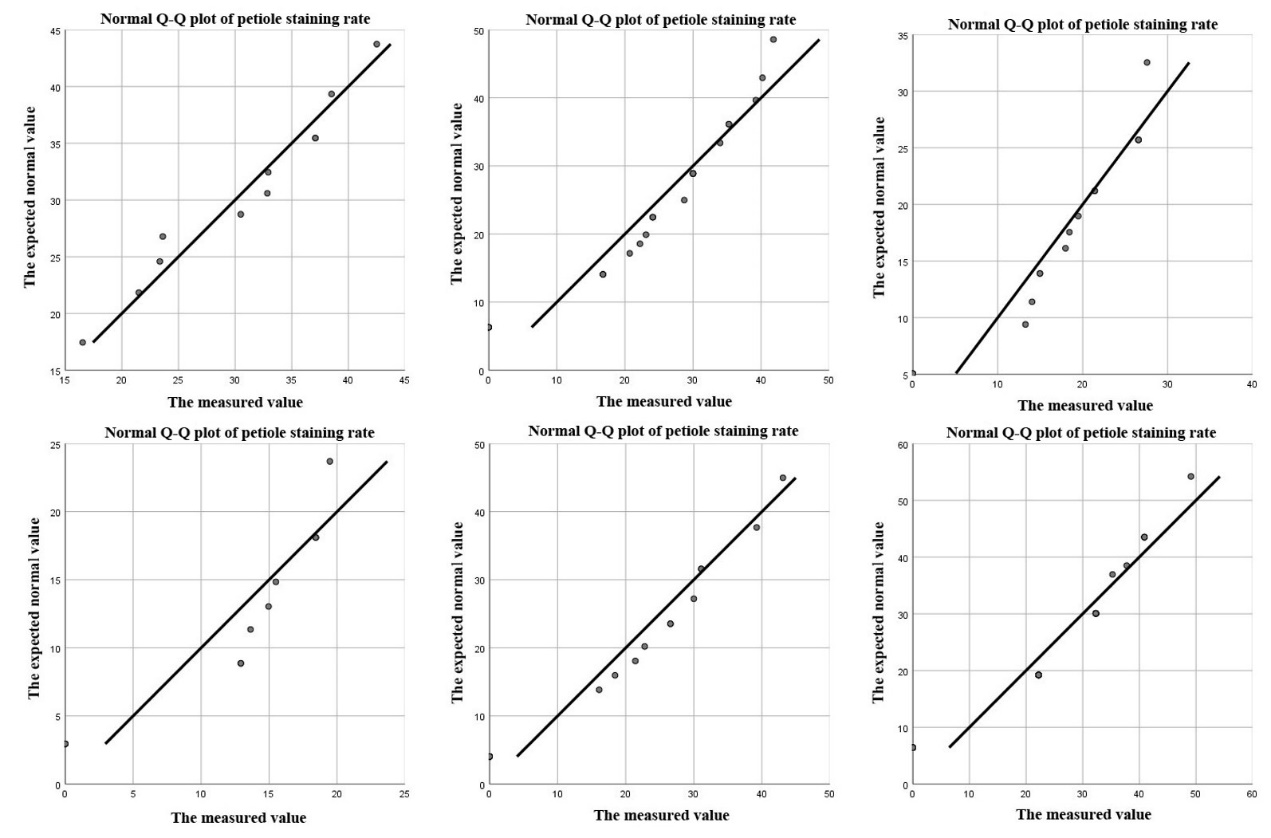


1. Q-Q plot of Supplementary Figure 1


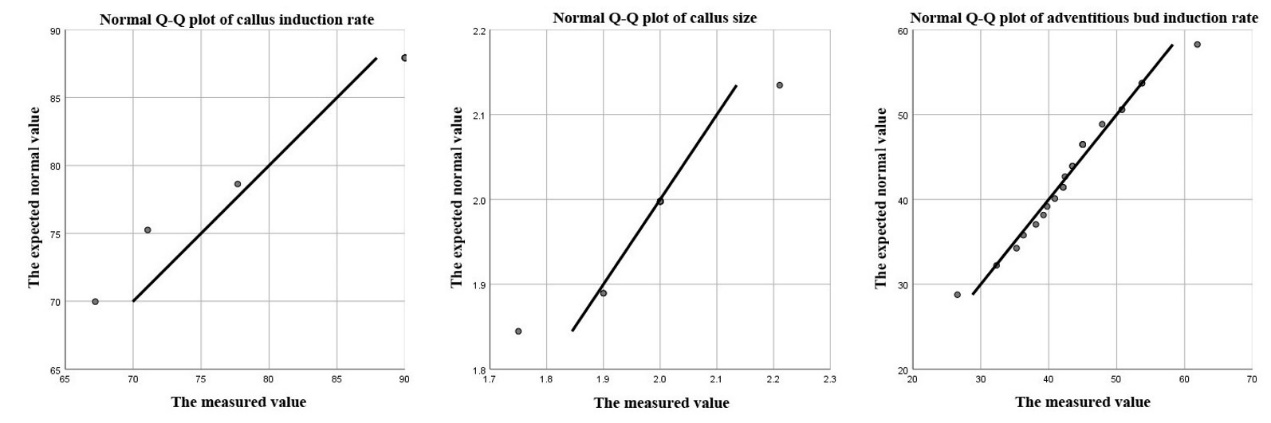


1. Q-Q plot of Supplementary Figure 2


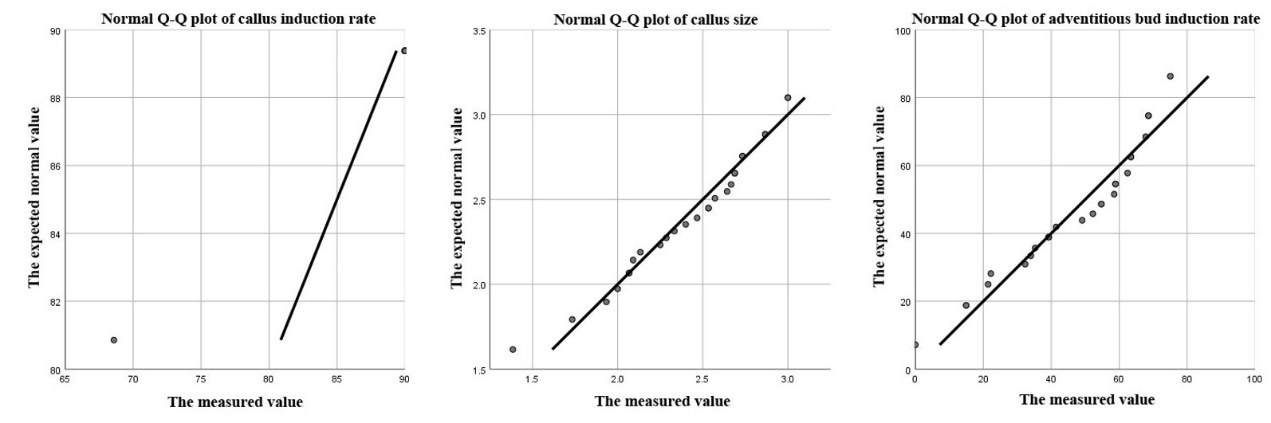


1. Q-Q plot of Supplementary Table 1


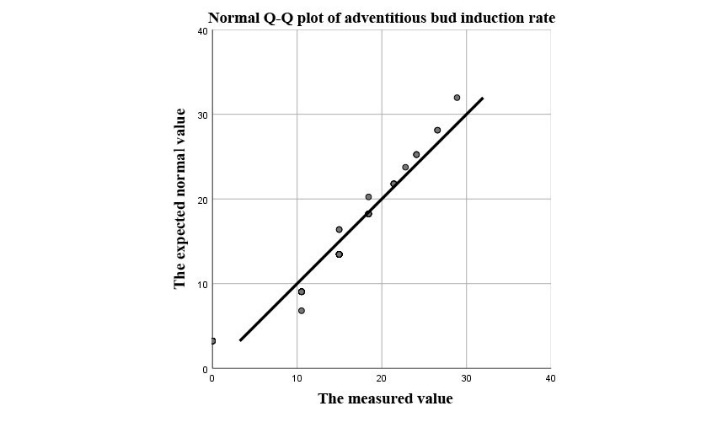


1. *P*-value

| Figure | *P*-value |
| --- | --- |
| Figure 1B | 0.044 |
| Figure 1C | 0.507 |
| Figure 1D | 0.000 |
| Figure 3A | 0.547 |
| Figure 3B | 0.065 |
| Figure 3C | 0.659 |
| Figure 3D | 0.026 |
| Figure 3E | 0.074 |
| Figure 4B | 0.191 |
| Figure 4C | 0.117 |
| Figure 4D | 0.000 |
| Figure 4E | 0.000 |
| Figure 4F | 0.001 |
| Figure 5B | 0.000 |
| Figure 5C | 0.005 |
| Figure 5D | 0.000 |
| Figure 5E | 0.000 |
| Figure 5F | 0.000 |
| Figure 6A Leaf staining rate | 0.733 |
| Petiole staining rate | 0.468 |
| Figure 6B Leaf staining rate | 0.000 |
| Petiole staining rate | 0.001 |
| Figure 6C Leaf staining rate | 0.143 |
| Petiole staining rate | 0.652 |
| Figure 6D Leaf staining rate | 0.001 |
| Petiole staining rate | 0.273 |
| Figure 6E Leaf staining rate | 0.004 |
| Petiole staining rate | 0.110 |
| Figure 6F Leaf staining rate | 0.599 |
| Petiole staining rate | 0.399 |
| Supplementary Figure 1A | 0.181 |
| Supplementary Figure 1B | 0.463 |
| Supplementary Figure 1C | 0.903 |
| Supplementary Figure 2A | 0.558 |
| Supplementary Figure 2B | 0.018 |
| Supplementary Figure 2C | 0.047 |
| Supplementary Table 1 | 0.001 |
