# Supplementary material for: Agrobacterium-mediated genetic transformation of the most widely cultivated superior clone Eucalyptus urophylla × E. grandis DH32-29 in Southern China
Source: Front Plant Sci. 2023 Jan 17;13:1011245. doi: 10.3389/fpls.2022.1011245 (PMC9886895; doi:10.3389/fpls.2022.1011245)
Supplement: Supplementary file 4 [file Table_1.docx]

Supplemental Table 1 Effects of plant growth regulators for regeneration induced by stem internodes as explants of *E. urophylla* × *E. grandis* DH32-29

| Treatment | NAA (mg·L^-1^) | CPPU (mg·L^-1^) | TDZ (mg·L^-1^) | Frequency of adventitious bud induction (%) |
| --- | --- | --- | --- | --- |
| CIM1 | 0.02 | 0.06 | － | 10.83 ± 2.50 ab |
| CIM2 | 0.02 | 0.12 | － | 10.00 ± 1.92 abc |
| CIM3 | 0.02 | 0.24 | － | 17.22 ± 3.09 a |
| CIM4 | 0.02 | － | 0.06 | 11.11 ± 1.11 ab |
| CIM5 | 0.02 | － | 0.12 | 11.33 ± 2.26 ab |
| CIM6 | 0.02 | － | 0.24 | 3.33 ± 1.49 d |
| CIM7 | 0.5 | 0.1 | － | 10.00 ± 3.33 abcd |
| CIM8 | 0.5 | － | 0.09 | 2.67 ± 1.25 cd |
| CIM9 | 0.02 | 0.36 | － | 10.00 ± 1.92 abc |
| CIM10 | 0.02 | 0.48 | － | 4.76 ± 1.60 bcd |
| CIM11 | 0.02 | 0.60 | － | 5.33 ± 1.70 bcd |
| CIM12 | 0.02 | 0.72 | － | 3.81 ± 1.53 bcd |
| CIM13 | 0.2 | 0.1 | － | 5.56 ± 1.11 abcd |
| CIM14 | 0.3 | 0.1 | － | 6.67 ± 0.00 abcd |
| CIM15 | 0.4 | 0.1 | － | 3.33 ± 1.92 bcd |
| CIM16 | 0.5 | 0.1 | － | 5.00 ± 1.67 bcd |
| CIM17 | 0.6 | 0.1 | － | 2.22 ± 2.22 d |

Different letters indicate significant differences among treatments using the Duncan’s multiple range test at *P* < 0.05.
